# Interventions to improve antiretroviral adherence in HIV-infected pregnant women: A systematic review and meta-analysis

**DOI:** 10.3389/fpubh.2022.1056915

**Published:** 2022-12-08

**Authors:** Jie Zhou, Jingyi Yun, Xinxin Ye, Wen Liu, Wenhan Xiao, Peige Song, Hongmei Wang

**Affiliations:** ^1^Department of Social Medicine of School of Public Health and Department of Pharmacy of the First Affiliated Hospital, Zhejiang University School of Medicine, Hangzhou, Zhejiang, China; ^2^Department of Social Medicine of School of Public Health and Women's Hospital, Zhejiang University School of Medicine, Hangzhou, China

**Keywords:** HIV, medication adherence, antiretroviral therapy, pregnant women, systematic review

## Abstract

**Background:**

Medication adherence in HIV-infected pregnant women remains suboptimal. This systematic review and meta-analysis aimed to evaluate the effectiveness of interventions on improving antiretroviral adherence targeting among HIV-infected pregnant women.

**Methods:**

Five databases were screened to identify quasi-experimental studies and randomized controlled trials. The risk ratios (*RR)* and confidential intervals (*CI*) were extracted to estimate the improvement in antiretroviral adherence after interventions compared with control conditions. This study was registered with PROSPERO, number CRD42021256317.

**Results:**

Nine studies were included in the review, totaling 2,900 participants. Three interventions had significance: enhanced standard of care (eSOC, RR 1.14, 95%CI 1.07–1.22, Z = 3.79, *P* < 0.01), eSOC with supporter (RR 1.12, 95%CI 1.04–1.20, Z = 2.97, *P* < 0.01) and device reminder (RR 1.33, 95%CI 1.04–1.72, Z = 2.23, *P* = 0.03).

**Discussion:**

The study supported the eSOC and the device reminder as effective intervention strategies for improving HIV medication adherence. Based on the current findings, the study called for more efforts to improve antiretroviral care for pregnant women through involving multicenter, large-sample, and high-quality research and combining the device reminder with other intervention methods.

**Systematic review registration:**

https://www.crd.york.ac.uk/prospero/display_record.php?ID=CRD42021256317, identifier CRD42021256317.

## Introduction

There are approximately 37.6 million people suffering from HIV globally. In 2020, more than half of the people living with HIV (PLWH) were in eastern and southern Africa, with females accounting for nearly 50% of new HIV infectors (1, 2). Owing to various Prevention of Mother-to-Child Transmission (PMTCT) programs, 95% of HIV-infected pregnant women living in eastern and southern Africa had access to antiretroviral medicines to reduce mother-to-child transmission of HIV. Despite emerging preventive programs worldwide, the HIV prevention services are less assessable in Eastern and Southern Africa (2–7). Moreover, poor adherence to medication was commonly found in HIV-infected pregnant women (8, 9). It was suggested that the medication adherence declined across the gestation, and was lower in the postnatal period compared to the prenatal period (10–15).

Multiple factors could lead to inadequate adherence to medication among pregnant women living with HIV, including depressive symptoms, financial dependence, morning sickness, social stigma, and caregiver burden (16–19). Additionally, in resource-shortage regions, such as South Africa, non-disclosure of HIV serostatus to life partners was significantly associated with poor adherence during pregnancy (20). To address such inadequate adherence, a series of interventions have been implemented and evaluated, such as incentives, short message service (SMS), supporters, and cognitive behavioral therapy (21–24). However, the evidence regarding their effectiveness of improving adherence remains mixed (25). For example, the enhanced standard of care (eSOC), as one of the primary interventions, is used to provide counseling or short educational sessions on medication adherence for pregnant women living with HIV. While no significant group difference in pharmacy adherence was found in a study based on video-viewing (25, 26). Moreover, measurements to evaluate medication adherence have been inconsistent (27). According to the evidence base, the pill count and self-report are the most commonly used among interventional studies (28). Meanwhile, several research used the medication events monitoring system (MEMS) as the outcome measurement, which records times of opening the container by using a microprocessor (29–31). As a result of measurement inconsistency, the barrier is created to comparison among different interventions.

Additionally, according to the 90-90-90 target, 90% of people living with HIV globally should be diagnosed, 90% of those diagnosed should receive antiretroviral therapy (ART) and 90% of them ought to achieve virological suppression (32). A meta-analysis evaluated the effectiveness of various interventions to improve adherence in pregnant women in sub-Saharan Africa. In their study, pregnant women who had not received medication before the intervention were included together with PMTCT program participants. As such, the denominator in calculating medication adherence rate was a mixture of HIV-infected pregnant women who chose to start to take medicine and PMTCT program participants, making it difficult to distinguish the effects of intervention itself and PMTCT. Besides, little is known about whether or how different study characteristics, including study design, setting, measurements used, and intervention duration, impact the effectiveness of the previous intervention to improve medication adherence among pregnant women. To fill these gaps of knowledge, the current meta-analysis sought to investigate the effectiveness of different interventions in medication adherence which may provide insights for better approaches to support pregnant women living with HIV.

## Methods

The Preferred Reporting Items for Systematic Reviews and Meta-Analyses (PRISMA) guideline was used for this systematic review and meta-analysis (33). This review was registered in the international prospective register of systematic reviews (registration number: CRD42021256317). Two reviewers conducted the review independently and disagreements between the two reviewers were resolved through discussing with a third reviewer.

### Search strategy and selection criteria

To reduce heterogeneity caused by the development of adherence intervention in recent years, we only included studies that were conducted in 2000s and therefore limited the publication date of articles to January 2000 till May 2021. Five databases were searched: Cochrane Central Register of Controlled Trials, PubMed, Embase, CINAHL, and Web of Science. We also updated the search of conference abstracts and non-article texts on the Bielefeld Academic Search Engine. The search terms were split into four components: HIV/AIDS, pregnancy, medication and adherence. A detailed search strategy is presented in Supplementary Table S1.

Studies that met the following criteria were included in this meta-analysis: (1) participants were HIV positive pregnant women only; (2) studies defined clear medication adherence measures; (3) studies were randomized controlled trials (RCTs) or quasi-experimental studies. Studies were excluded if they reported a medication initiation ratio improved by the prevention of transmission program instead of medication adherence outcome.

Researchers (JZ and JYY) independently reviewed all titles and abstracts after duplications had been removed and then conducted a full-text review following the inclusion and exclusion criteria. A third investigator (XXY) participated in the discussion regarding the eligibility of studies when any discrepancies occurred.

Data extraction for the included studies was completed using a standardized extraction form. The following data were included: (1) study characteristics (e.g., first author, publication year, country, and study design); (2) demographics of the study sample (e.g., age and sample size); (3) intervention approaches, measures of adherence, and thresholds of adherence. The definition of adherence was the proportion of women adherent to medication in the intervention and control groups (34). Adherence data from each article has been dual reviewed by JZ and JYY and then transferred into absolute number format. Discrepancies were consulted with the third investigator (XXY).

The quality of the included RCTs and quasi-experimental studies was assessed based on the Cochrane guideline (35). An overall score of study quality was first assigned and then the quality of reporting, internal validity (bias and confounding), power and external validity were marked by two researchers independently. All marks were classified into three levels, including (1) level A: less than or equal to three unsatisfied criteria; (2) level B: greater than three but less than seven unsatisfied criteria; (3) level C: greater than or equal to seven unsatisfied criteria. There are five dimensions of quality evaluation based on the Grading of Recommendations Assessment, Development, and Evaluation (GRADE) system: the risk of bias, inaccuracy, inconsistency, indirectness, and publication bias (36–38). GRADEpro software was used in this process and the quality of the evidence was categorized into four levels to reflect the strength of evidence: high quality, medium quality, low quality, and very low quality.

### Data analysis

The extracted data were exported to Review Manager 5.4 software for meta-analysis. Risk ratios (RRs) and 95% confidence intervals (CIs) were used to quantify the association between intervention effect and adherence to medication. Since intervention was used as “exposure,” it could be interpreted as a protective factor if RR and corresponding 95% CI are above 1.

Heterogeneity was assessed using the *I*^2^ statistic. Heterogeneity refers to the variation between the included studies assessed as follows: if *I*^2^ ≤ 49%, this was considered “low” heterogeneity; if *I*^2^ = 50 − 74%, this was considered “moderate” heterogeneity; and if *I*^2^ ≥ 75%, this was considered “high” heterogeneity (39). Subgroup analyses were conducted to investigate the extent to which study characteristics and adherence measures may have been potential sources of heterogeneity. A random-effects model was chosen when considerable heterogeneity was found within a subgroup. A funnel plot was used in reporting publication bias. The number of included studies in our meta-analysis is <10, which means the result of regression may be unstable, in this case, the results of meta-regression were presented in supplement (40).

## Results

**Figure 1:**
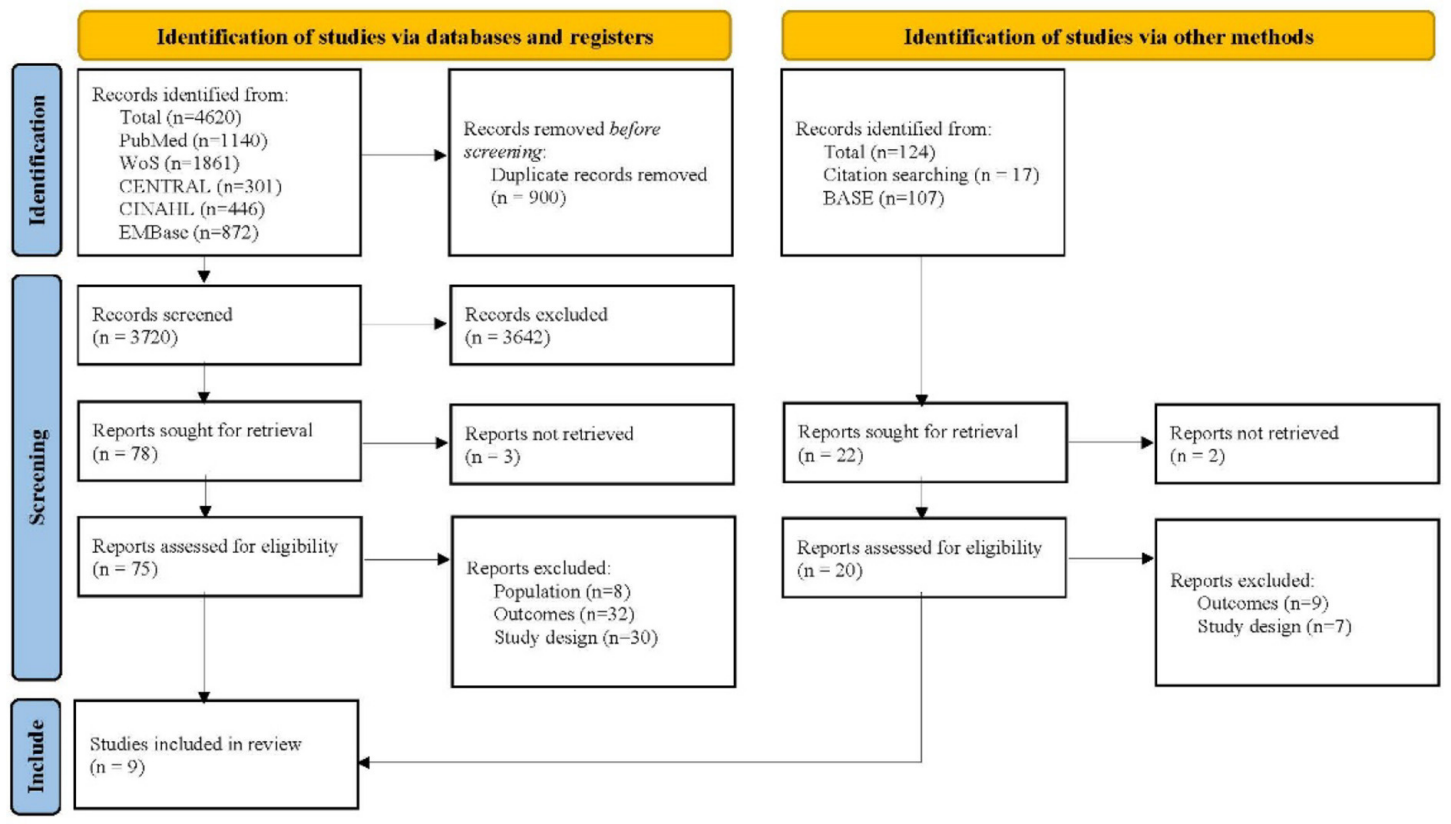
Flowchart of study selection.

The database search yielded 4,620 records and the citation search yielded 17 records. The 17 studies retrieved in the citation search came from a systematic review exploring the similar topic. After removing 900 duplicate studies, the remaining records' titles and abstracts were reviewed. Of these, 3,642 were found to be irrelevant and were therefore excluded. Then two researchers independently reviewed the full texts of 75 studies retrieved from the database search and 15 reports from the citation search. With 81 studies excluding due to the unmet population, study design, and outcome, a total of nine studies were finally included in this meta-analysis (Figure 1) (22, 23, 26, 41–46).

### Study characteristics

The nine selected studies including 2,900 HIV-positive participants were published between 2011 and 2020. The main characteristics of each study are shown in Table 1. Except for one study conducted in China, the rest included studies were all from Africa. In terms of study design, the included studies comprised seven randomized controlled trials and two quasi-experimental studies. Five of these studies were conducted in urban settings, three were conducted in rural settings and one was conducted in both urban and rural settings. Six studies reported demographics showed that the mean age of participants was under 30 years. For the outcomes measurement, five of the studies collected adherence data by counting the remaining pills, two used dried blood spots (DBS), one used medication possession ratio (MPR), and one used electronic monitoring systems (EMS). Participants from those studies were considered adherent to medication according to each standard of studies with the adherence threshold of at least 90%. The other two studies used DBS, which adapted the detection of antiretroviral drugs in a blood sample as the measurement of adherence.

**Table 1:** Characteristics of studies included (*N* = 9).

**References**	**Country**	**Setting**	**Study design**	**Sample size**	**Age** **mean ±SD**	**Intervention**	**Length of time (m)**	**Description of intervention methods**	**ADH measure^a^**	**THD of ADH^b^, %**
Cheng et al. (46)	China	Urban	Randomized controlled trial	60	26.0	Device reminder	3	Participants used an intelligent electronic medicine kit that can help patients remind them to take drugs constantly.	Electronic monitoring systems	>95%
Kieffer et al. (45)	Swaziland	Urban and rural	Quasi-experimental study	922	NR^c^	eSOC^d^	4	Participants received care by nurses who had a 1-day training course which was provided to increase knowledge and skills in provision of PMTCT and to enhance confidence and skills in counseling.	Dried blood spots	detection of NVP
Kim et al. (26)	Malawi	Urban	Randomized controlled trial	306	27.4	eSOC^d^	1	Participants received VITAL (Video-intervention to Inspire Treatment Adherence for Life) before ART.	Pill count	≥90%
Kiweewa et al. (23)	Uganda	Rural	Randomized controlled trial	85	27.3 ± 5.2	Supporter	12	ART nurses managed most follow-up visits at longer intervals between visits, and patients were supported by peer counselors and home visiting.	Pill count	≥95%
Mepham et al. (44)	South Africa, Kenya, and Burkina Faso	Rural	Randomized controlled trial	94	NR	eSOC^d^	9	Adherence counseling carried out by pharmacy staff and additional adherence counseling was provided by Zulu interviewers experienced in adherence counseling and with similar social backgrounds to mothers.	Pill count	≥95%
Nance et al. (41)	Tanzania	Urban	Cluster randomized controlled trial	678	NR	eSOC^d^ with supporter	11	Participants received interventions with four integrated components: (1) formal linkage of CHWs to health facilities; (2) CHW-led antiretroviral therapy (ART) adherence counseling; (3) loss to follow-up tracing by CHWs; and 4) Action Birth Cards (ABCs), a birth planning tool.	Medication possession ratio	≥95%
Okonji et al. (43)	Kenya	Urban	Quasi-experimental study	434	24.0 (21.0–27.0)^d^	eSOC^d^ with supporter	6	The status of participants between 14 weeks and 24 weeks postpartum who received adherence counseling and social support from study staff.	Pill count	≥95%
Weiss et al. (42)	South Africa	Rural	Randomized controlled trial	24	28.2 ± 7.1	eSOC^d^	1	Participants took part in 4 successive sessions utilized a cognitive-behavioral training approach, which were led by trained gender-matched facilitators.	Dried blood spots	detection of antiretroviral drugs
Yotebieng et al. (22)	Congo	Urban	Randomized controlled trial	297	28.5 (25.0–34.0)^d^	Incentives	17	Participants received standard of care plus small and increasing cash payments.	Pill count	100%

a ADH measure, adherence measure;

b THD of ADH, threshold of adherence;

c NR, not reported;

d eSOC, enhanced standard of care; ^e^median age and interquartile range.

### Intervention characteristics

The included studies evaluated four medication adherence-improving interventions and one mixed intervention. Our present study referred to the following definitions described by Kanters et al. for intervention classification: (1) enhanced standard of care (eSOC), which is a commonly used intervention including adherence counseling and group sessions aiming at increasing participants' knowledge of ART as well as motivation to adopt ART; (2) supporter, defined as any kind of support from peer, family, health educator or other individuals; (3) incentive, which means the use of material or financial reward; (4) device reminder, referring to interventions that use an alarm clock, electronic medicine kit, or other devices to manage medication intake (47).

### Quality assessment

Quality assessment scores of included studies are shown in Table 2. Four of the nine studies were rated as level A, and five of them were rated as level B. No study was rated as level C. All studies recruited participants from representative samples.

**Table 2:** Quality scores for assessing the risk of bias in the RCTs and quasi-experimental study.

**References**	**Random allocation sequence generation method^a^**	**Random sequence hiding^b^**	**Blinding for patients and researchers^b^**	**Blinding for outcome evaluator^b^**	**Selective report outcome^b^**	**Lost follow-up/ drop-out/ exit^b^**	**Other biases^b^**	**Baseline data^c^**	**intention-to-treat analysis^b^**	**Level**
Kieffer et al. (45)	4	2	0	0	2	1	1	0	1	A
Mepham et al. (44)	4	2	2	2	0	0	2	0	2	B
Okonji et al. (43)	4	2	0	0	0	1	2	0	2	B
Weiss et al. (42)	2	0	0	0	0	1	1	1	2	B
Kiweewa et al. (23)	1	1	0	0	0	0	0	1	2	A
Yotebieng et al. (22)	4	2	0	0	0	1	1	1	2	A
Nance et al. (41)	3	0	0	0	0	1	0	1	1	B
Kim et al. (26)	4	0	0	0	0	0	0	1	2	A
Cheng et al. (46)	1	2	2	2	0	0	0	0	0	B

a 0 = no, 1 = table from random number, 2 = computer generated, 3 = block randomization, 4 = not clear;

b 0 = no, 1 = yes, 2 = not clear;

c 0 = no description, 1 = comparable.

### Main analysis

**Figure 2:**
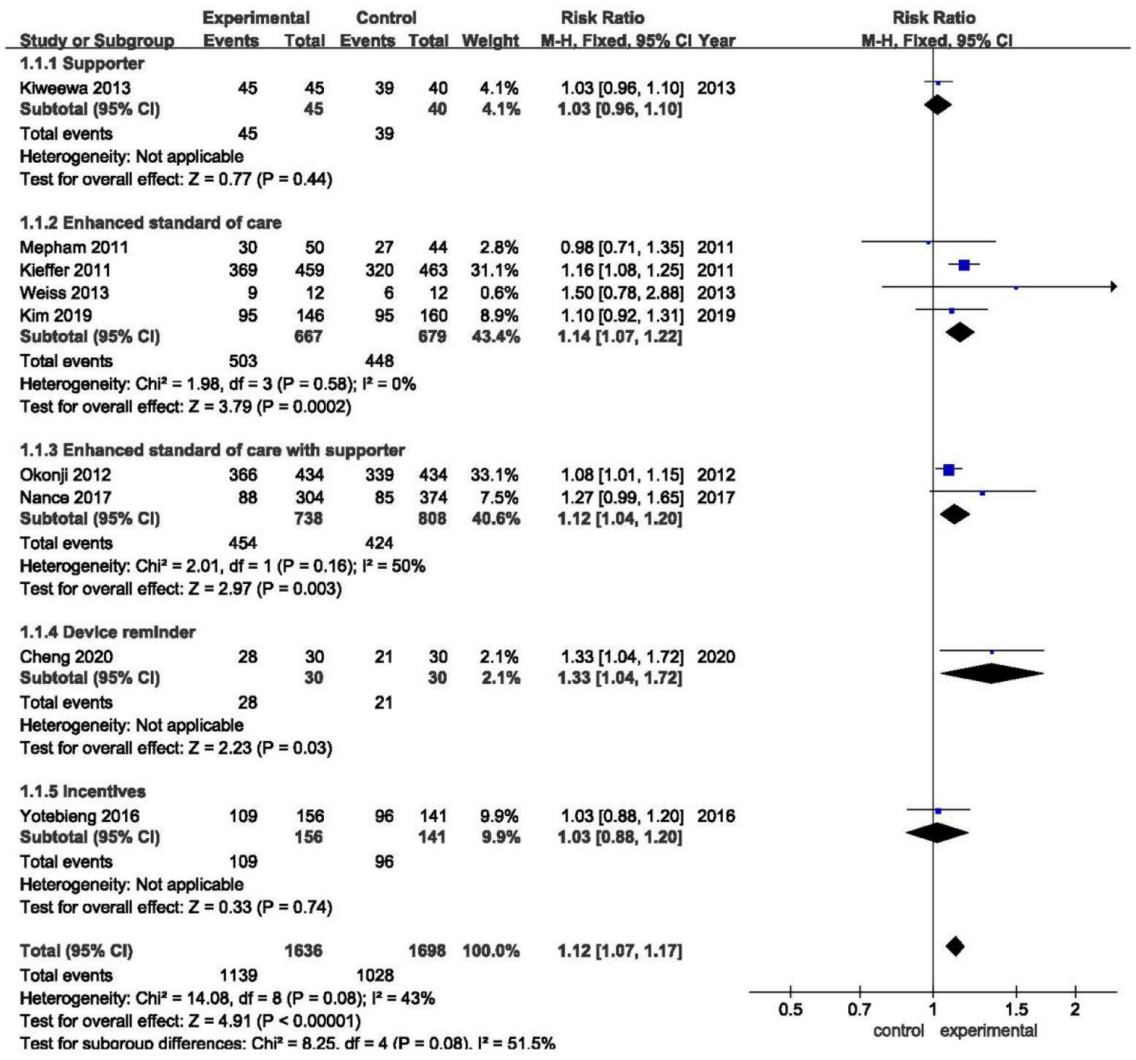
Overall forest plot showing the impact of different improvement approaches to medication adherence.

Figure 2 shows the overall effect sizes for studies that reported the impact of five approaches to improve the adherence to medication of pregnant women living with HIV. Of the total participants analyzed, 1,139 of 1,636 (69.6%) in intervention groups and 1,028 of 1,698 (60.5%) in control groups had good medication adherence (RR 1.11, 95%CI 1.07–1.17, Z = 4.91, *P* < 0.01). In our meta-analysis, three interventions that made a significant impact were: eSOC (RR 1.14, 95%CI 1.07–1.22, Z = 3.79, *P* < 0.01), eSOC with supporter (RR 1.12, 95%CI 1.04–1.20, Z = 2.97, *P* < 0.01), and device reminder (RR 1.33, 95%CI 1.04–1.72, Z = 2.23, *P* = 0.03). However, there were no statistically significant differences in supporter (RR 1.03, 95%CI 0.96–1.10, Z = 0.77, *P* = 0.44) and incentives (RR 1.03, 95%CI 0.88–1.20, Z = 0.33, *P* = 0.74) as compared with control groups.

The overall *I*^2^ was 43.0%, while after being stratified into five subgroups, the heterogeneity within the enhanced standard of care group had dramatically decreased (*I*^2^ = 0%).

### Subgroup analysis

The results of subgroup analyses are shown in Table 3. The subgroup analyses by study design showed the both significant improvement results for quasi-experimental studies (RR 1.12, 95%CI 1.04–1.20, Z = 2.96, *P* < 0.01) and RCT (RR 1.12, 95%CI 1.02–1.22, Z = 2.45, *P* = 0.01). Moreover, the similarly positive results were seen from the following subgroup meta–analyses: firstly, significant changes were obtained while measured by DBS (RR 1.17, 95%CI 1.08–1.26, Z = 4.06, *P* < 0.01), pill count (RR 1.05, 95%CI 1.01–1.09, Z = 2.27, *P* = 0.02), and EMS (RR 1.12, 95%CI 1.07–1.17, Z = 2.23, *P* = 0.03); secondly, interventions were found to be effective while conducted in urban (RR 1.10, 95%CI 1.03–1.18, Z = 2.74, *P* < 0.01), urban and rural (RR 1.16, 95%CI 1.08–1.23, Z = 3.91, *P* < 0.01); positive effect were also found in different region/country, Africa (RR 1.11, 95%CI 1.06–1.17, Z = 4.67, *P* < 0.01), China (RR 1.33, 95%CI 1.04–1.72, Z = 2.23, *P* = 0.03); thirdly, as for intervention duration, intervention < 6 months was tested to be significantly effective (RR 1.16, 95%CI 1.07–1.17, Z = 4.09, *P* < 0.01). Finally, with the development of the management package for HIV/AIDS, comprehensive clinical care has been promoted worldwide (48, 49). Therefore, the basic educational conditions of earlier studies may differ from recent trials and further analysis support that time–varying may result in heterogeneity: before 2015 (RR 1.09, 95%CI 1.01–1.17, Z = 2.25, *P* = 0.02), 2015 and later (RR 1.14, 95%CI 1.02–1.26, Z = 2.38, *P* = 0.02). Although *I*^2^ of study setting and measurement were low, variations within quasi–experimental study and high GRADE level subgroups were even higher than the total heterogeneity.

**Table 3:** Subgroup analyses by study characteristics.

**Subgroup**	***n***	**Statistical method**	**Risk ratio** **(95% CI)**	**Z**	***P***	**I^2^ (%)**
Overall	9	Mean difference (M-H, fixed, 95% CI)	**1.12 (1.07, 1.17)**	**4.91**	**< 0.01**	43
**Adherence measures**						
Dried blood spots	2	Mean difference (M-H, fixed, 95% CI)	**1.17 (1.08, 1.26)**	**4.06**	**< 0.01**	0.0
Pill count	5	Mean difference (M-H, fixed, 95% CI)	**1.06 (1.01, 1.12)**	**2.27**	**0.02**	0.0
Medication possession ratio	1	Mean difference (M-H, fixed, 95% CI)	1.27 (0.99, 1.65)	1.85	0.06	/
Electronic monitoring systems	1	Mean difference (M-H, fixed, 95% CI)	**1.12 (1.07, 1.17)**	**2.23**	**0.03**	/
**Study setting**						
Urban	5	Mean difference (M-H, fixed, 95% CI)	**1.10 (1.03, 1.18)**	**2.74**	**< 0.01**	15
Urban and rural	1	Mean difference (M-H, fixed, 95% CI)	**1.16 (1.08, 1.25)**	**3.91**	**< 0.01**	/
Rural	3	Mean difference (M-H, fixed, 95% CI)	1.03 (0.96, 1.10)	0.85	0.40	0
**Region/country**						
Africa	8	Mean difference (M-H, fixed, 95% CI)	**1.11 (1.06, 1.17)**	**4.67**	**< 0.01**	39
China	1	Mean difference (M-H, fixed, 95% CI)	**1.33 (1.04, 1.72)**	**2.23**	**0.03**	/
**Study design**						
Quasi experimental design	2	Mean difference (M-H, random, 95% CI)	**1.12 (1.04, 1.20)**	**2.96**	**< 0.01**	55
Randomized controlled trial	7	Mean difference (M-H, fixed, 95% CI)	**1.12 (1.02, 1.22)**	**2.45**	**0.01**	48
**Length of intervention**						
≤ 6 months	5	Mean difference (M-H, fixed, 95% CI)	**1.13 (1.07, 1.18)**	**4.90**	**< 0.01**	18
> 6 months	4	Mean difference (M-H, random, 95% CI)	1.06 (0.93, 1.19)	0.88	0.38	52
**Published year**						
Before 2015	5	Mean difference (M-H, random, 95% CI)	**1.09 (1.01, 1.17)**	**2.25**	**0.05**	56
2015 and later	4	Mean difference (M-H, fixed, 95% CI)	**1.14 (1.02, 1.26)**	**2.38**	**0.02**	28

The bold values indicate the values which are statistically significant at the level of 0.05.

### GRADE evidence of outcomes

**Figure 3:**
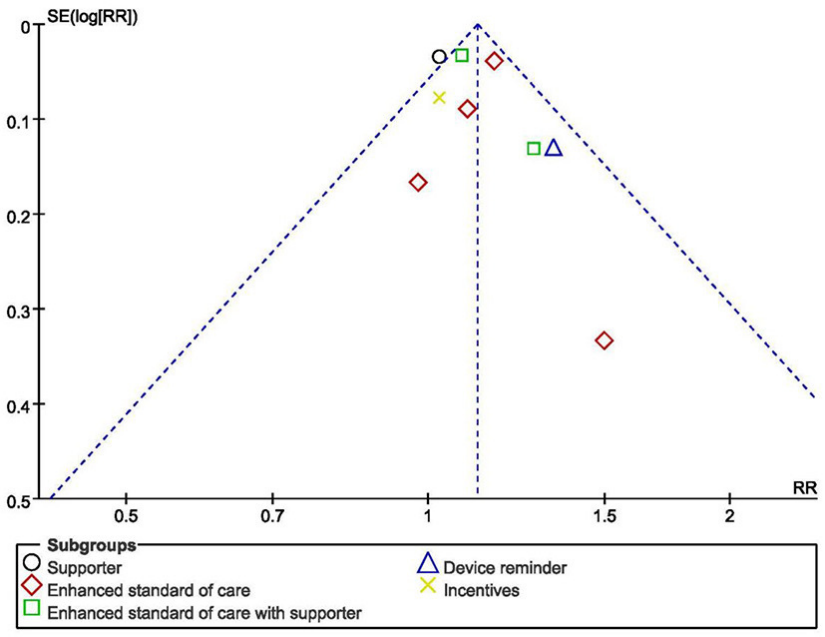
Funnel plot showing publication bias for studies reporting impact on adherence to medication of HIV+ pregnant women.

The risk of bias might exist because of the method of randomization, allocation concealment and unspecified blinding (50). The overall quality of the evidence for four adherence outcomes was moderate to high, but the results were considered low for viral load and CD4 count (Table 4). The funnel plot showed an even scattering of points around the central axis, indicating no publication bias (Figure 3).

**Table 4:** GRADE evidence profile of outcomes.

**Certainty assessment**							**No. of patients**		**Effect**		**Certainty**
**Outcomes**	**No.of studies**	**Risk of bias**	**Inconsistency**	**Indirectness**	**Imprecision**	**Publication bias**	**INV^a^**	**CON^b^**	**RR/SMD**	**95%CI**	
DBS	2	Not serious	Not serious	Not serious	Not serious	Undetected	1,230	1,238	0.98	[0.97, 0.99]	High
Pill count	5	Not serious	Serious	Not serious	Serious	Undetected	875	869	1.11	[1.05, 1.18]	Moderate
Viral load	3	Not serious	Not serious	Not serious	Serious	Undetected	424	363	1.03	[0.99, 1.07]	Low
Medication possession ratio	1	Not serious	Not serious	Not serious	Serious	Undetected	304	374	1.27	[0.99, 1.65]	Moderate
CD4 count	2	Not serious	Very serious	Not serious	Not serious	Undetected	70	75	−9.82	[-12.45,-7.19]	Low
Electronic monitoring systems	1	Not serious	Not serious	Not serious	Not serious	Undetected	30	30	1.33	[1.04, 1.72]	High

a INV = intervention;

b CON = control; ^c^CI = confidence interval; ^d^SMD = standard mean difference; ^e^DBS = dried blood spots; ^f^RR = relative ratio.

## Discussion

In this study, nine trials assessing the effect of interventions on improving medication adherence among pregnant women with HIV were analyzed. These nine trials that included 2,900 participants were conducted primarily in low- and middle-income countries (LMICs) across Africa, except one RCT conducted in Hangzhou, the capital of Zhejiang province, China. No data from high-income countries has been found.

This meta-analysis found that eSOC, eSOC with supporter, and device reminders positively improved medication adherence among pregnant HIV-positive women compared with standard care conditions. Pregnant women living with HIV are particularly vulnerable, with many factors influencing their medication-taking behavior (13, 16). It has been found that poor knowledge of HIV/AIDS amongst pregnant women with HIV is associated with high transmission and delay in health-seeking behavior (49). Therefore, the eSOC intervention has been adapted into many PMTCT programs in order to raise awareness of pregnant women regarding HIV treatment (51–53). Previous evidence has also shown that eSOC was able to help these women access to social support and further increase the medication adherence rate (25, 54). Nevertheless, eSOC has been deemed unsustainable in LMICs due to a lack of trained professionals (47). In contrast to the previous meta-analysis, our study suggested that the combination of social network support and education counseling is sufficient to guide pregnant women to better adherence against HIV (25), thereby, it may helpfully provide alternative measures in those countries. This difference might be related to the study design and measurement of medication adherence as we included RCTs and quasi-experimental studies where upscaling programs based on eSOC are normally absent. However, we found that the effect of eSOC combined with supporter is even lower than single intervention. It may be explained by the fact that visits took by doctors or peer counselors during study was the major intervention, so the effect was possibly obscured with prenatal care provided by doctors or family members.

Our study also suggested that device reminder might be particularly suitable for pregnant women living with HIV in developing countries. It is one of the most cost-effective interventions in the long run, however, it requires economic input at the very beginning. Previous evidence showed that device reminder including clocks, smart pillboxes, and other types of equipment that could ring or flash at a time set by users were able to reduce the incidence of forgetting to take pills, which has been considered as a major barrier of ART medication adherence (55, 56). In addition, with the social isolation and nondisclosure of HIV, physical and mental stress were identified as barriers to HIV therapy adherence (57). In China, pregnant women with HIV/AIDS have lower social support and those who were not disclosing HIV status to anyone other than health care providers showed a greater willingness to receive reminders than others (54). These results suggested that device reminders may be more effective with people live without enough social support, and, more suitable for regions that could afford for the device brought into service but hardly increase health care workers (58). Overall, our evidence indicated that device reminders is an effective intervention and may be better to combine with SMS, particularly for pregnant women in LMICs. Furthermore, for the future clinical application, peer counselors, mobile phone text messages and other interventions are also emphasized by WHO's guidance on adherence support programs (59). Future research should explore how partner and community support combined with device reminder can be harnessed to improve intervention effectiveness.

Subgroup analyses showed that intervention effects differed based on study design, region, outcome measurements and intervention duration. Moreover, the homogeneity across subgroups indicated that geographic areas and measurement approaches of research could be confounders in trials. Firstly, the differences of intervention effect between regions of studies may lie in their differentiated economic conditions and the differences also can be explained by the diverse cultural background of different continents, since the perceived stress of interpersonal relationship with colleagues and even strangers was identified as one major psychological factor of HIV-infected women in China (60, 61). Although the vertical transmission rates were relatively low in high-income countries or areas, the medication adherence rates remained suboptimal reflecting by the aspects of viral suppression and drug resistance prevention, where pregnancy women with HIV and their children might be facing great challenges in preventing potential transmission and maintaining long-term life qualities (11, 12, 62). Therefore, it is crucial for high-income countries or regions to facilitate more ART adherence interventions during pregnancy and postpartum. Additionally, the durations of trials in this meta-analysis were from 1–17 months. In line with the previous systematic review and meta-analysis (47), the alteration from interventions were slight and seemed to wane over time after interventions were withdrawn. Since antiretroviral therapy is a lifelong requirement of PLWH, long-term and long-lasting interventions are still needed for women at high risk of inadequate adherence, especially during the postpartum period.

With regard to outcome measurements, all nine studies using different measurements of medication adherence among pregnant women might overestimate or underestimate the results (63). For example, pill count referring to the number of dosage units calculated between clinic visits can underestimates adherence because patients often refill their medication before running out (27, 64). Measuring medication drug levels through DBS has also been proven equally effective and more patient-friendly than plasma (65). Such direct measures are considered the most accurate, but they can only show whether patients have taken pills rather than reveal the detailed patterns of nonadherence. The other measurement like MPR, which was defined as the proportion of the daily supply obtained over refill interval, is acceptable and easy to calculate. However, the negligence of gaps in refills can lead to an overestimation in adherence (64, 66). Lastly, as for the commonly used self-report method, it is a subjective method with the advantages of low cost and simplicity (67). Given these reasons, future studies should have more careful consideration in selecting suitable and comparable measurements, and then strive for intervention and measurement consistency across economies.

While looking into the quality and potential bias of included studies. Due to the privacy and particularity of the disease, most of the included studies reported that the outcome assessors were not blinded to the exposure status of participants, which could lead to measurement bias. Allocation concealment occurred in all of the studies, for example, in forms of sequentially numbered opaque sealed envelopes, numbered or coded containers, central randomization by a coordinating center, and computer-generated randomization that is not revealed ahead of time. These methods have effectively reduced the selection bias. “Dropouts” refers to individuals for whom there are no endpoint measurements, often because they dropped out of the study or were lost to follow-up (68). In this meta-analysis, 20% loss to follow-up or below was considered to have no impact on the quality of evidence. The outcome indicators of the included studies are consistent with the purpose of their studies. Lastly, the funnel plot showed an even scattering of points along the central axis, indicating no publication bias.

This meta-analysis has several strengths besides updating the current evidence. First of all, it is the first meta-analysis that used more strict inclusion criteria of adherence outcome measurements and data format, which ensured a high quality of studies in the final analysis. The second, we did not set geographical limitations before retrieval, thereby, this study added multiplex experiences to previous studies that assessed intervention effectiveness within a particular country context.

Despite these strengths, this review has three main limitations. First, intervention effectiveness during the postnatal period was not assessed since none of these studies collected data from prenatal and postnatal periods separately. Secondly, nearly all of the included studies took place in African countries, with the exception of one which was conducted in China. Because of this, the results may not be generalizable to other settings and population. Finally, the inclusion of quasi-experimental studies might have introduced some confounds attributed to the lack of experimental control. Similarly, the variation in interventions, approaches and thresholds of outcome measurements might have influenced the pooled effect size of the intervention.

## Conclusions

This meta-analysis systematically reviewed studies published up to May 2021 that assessed the effect of interventions on medication adherence among HIV-positive pregnant women. The eSOC and device reminders were found to significantly improve adherence to medication. The results implied that comparing to the resource-intensive eSOC, device reminders are cost-effective and therefore may be most suitable for enhancing adherence among pregnant women with HIV in low-resource settings. Future research can be conducted to investigate the implementation of device reminder interventions in diverse contexts and its integration to the social support systems. Current results also highlighted the future efforts to improve antiretroviral care for pregnant women involve multicenter, large sample, and high-quality studies that use objective measures of adherence. In the end, this study called for more high-quality evidence in this topic, especially from developed countries, to inform clinical and political decision-making which can ultimately improve health outcomes for pregnant women living with HIV worldwide.

## Data availability statement

The original contributions presented in the study are included in the article/Supplementary material, further inquiries can be directed to the corresponding author/s.

## Author contributions

JZ and JY designed the study, conducted the literature searches, analyzed the data, and drafted the manuscript. XY, PS, WL, and HW provided input to the design, analysis, and edited the manuscript. All authors have read and approved the final version.

## References

[1] HIV/AIDS. Available online at: https://www.who.int/news-room/fact-sheets/detail/hiv-aids (accessed January 22, 2022).

[2] UNAIDS. Global AIDS Update. Available online at: https://www.unaids.org/sites/default/files/media_asset/UNAIDS_FactSheet_en.pdf (accessed January 22, 2022).

[3] Peltzer K Abbamonte JM Mandell LN Rodriguez VJ Lee TK Weiss SM . The effect of male involvement and a prevention of mother-to-child transmission (PMTCT) intervention on depressive symptoms in perinatal HIV-infected rural South African women. Arch Womens Ment Health. (2020) 23:101–11. 10.1007/s00737-019-00955-7 30798376 PMC6707893

[4] Newman OM Bellare NB Chakanyuka MCC Oyelade TA Thom EM Bigirimana F . Building health system capacity through implementation research: experience of INSPIRE-a multi-country PMTCT implementation research project. J Acquir Immune Defic Syndr. (2017) 75:S240–S7. 10.1097/QAI.0000000000001370 28498195 PMC5432100

[5] Girma M Wendaferash R Shibru H Berhane Y Hoelscher M Kroidl A. Uptake and performance of prevention of mother-to-child transmission and early infant diagnosis in pregnant HIV-infected women and their exposed infants at seven health centres in Addis Ababa, Ethiopia. Trop Med Int Health. (2017) 22:765–75. 10.1111/tmi.12881 28407452

[6] Herlihy JM Hamomba L Bonawitz R Goggin CE Sambambi K Mwale J . Implementation and operational research: integration of PMTCT and antenatal services improves combination antiretroviral therapy uptake for HIV-positive pregnant women in southern zambia: a prototype for option B+? J Acquir Immune Defic Syndr. (2015) 70:e123–9. 10.1097/QAI.0000000000000760 26181813 PMC6754251

[7] Ayuo P Musick B Liu H Braitstein P Nyandiko W Otieno-Nyunya B . Frequency and factors associated with adherence to and completion of combination antiretroviral therapy for prevention of mother to child transmission in western Kenya. J Int AIDS Soc. (2013) 16:17994. 10.7448/IAS.16.1.17994 23336727 PMC3536941

[8] Mirochnick M. Antiretroviral pharmacology in pregnant women and their newborns. Ann N Y Acad Sci. (2000) 918:287–97. 10.1111/j.1749-6632.2000.tb05498.x 11131716

[9] Laine C Newschaffer CJ Zhang D Cosler L Hauck WW Turner BJ. Adherence to antiretroviral therapy by pregnant women infected with human immunodeficiency virus: a pharmacy claims-based analysis. Obstet Gynecol. (2000) 95:167–73. 10.1097/00006250-200002000-00001 10674574

[10] Haas AD Msukwa MT Egger M Tenthani L Tweya H Jahn A . Adherence to antiretroviral therapy during and after pregnancy: cohort study on women receiving care in malawi's option B plus program. Clin Infect Dis. (2016) 63:1227–35. 10.1093/cid/ciw500 27461920 PMC5064160

[11] Nachega JB Uthman OA Anderson J Peltzer K Wampold S Cotton MF . Adherence to antiretroviral therapy during and after pregnancy in low-income, middle-income, and high-income countries: a systematic review and meta-analysis. Aids. (2012) 26:2039–52. 10.1097/QAD.0b013e328359590f 22951634 PMC5061936

[12] Zhou H Liu L Zhang M Chen X Huang Z. Antiretroviral therapy among pregnant and postpartum women in China: a systematic review and meta-analysis. Am J Infect Control. (2016) 44:E25–35. 10.1016/j.ajic.2015.10.034 26739641

[13] Mellins CA Chu C Malee K Allison S Smith R Harris L . Adherence to antiretroviral treatment among pregnant and postpartum HIV-infected women. AIDS Care - Psychol Socio-Med Asp. (2008) 20:958–68. 10.1080/09540120701767208 18608073

[14] Bardeguez AD Lindsey JC Shannon M Tuomala RE Cohn SE Smith E . Adherence to antiretrovirals among US women during and after pregnancy. JAIDS. (2008) 48:408–17. 10.1097/QAI.0b013e31817bbe80 18614923 PMC2764488

[15] Li. C Yujuan. Z Jiannv. H Chengjing. T Ying. ZS Qiuquan. J . Compliance of antiviral treatment and its correlation with pregnant outcomes in pregnant women with HIV infection in Hangzhou. Chin J Clini Infect Dis. (2020) 1. Available online at: https://www.webofscience.com/wos/alldb/full-record/CSCD:6676956

[16] Tuthill EL Maltby AE Odhiambo BC Akama E Pellowski JA Cohen CR . “I found out I was pregnant, and I started feeling stressed”: a longitudinal qualitative perspective of mental health experiences among perinatal women living with HIV. AIDS Behav. (2021) 25:4154–68. 10.1007/s10461-021-03283-z 33997940 PMC8126180

[17] Sarna A Singh RJ Duggal M Chandra P Reynolds N. The prevalence and determinants of depression among HIV-positive perinatal women receiving antiretroviral therapy in India. Arch Womens Mental Health. (2019) 22:399–404. 10.1007/s00737-018-0904-4 30141027 PMC6387642

[18] Adeniyi OV Ajayi AI Ter Goon D Owolabi EO Eboh A Lambert J. Factors affecting adherence to antiretroviral therapy among pregnant women in the Eastern Cape, South Africa. BMC Infect Dis. (2018) 18:175. 10.1186/s12879-018-3087-8 29653510 PMC5899366

[19] Holstad MM Spangler S Higgins M Dalmida SG Sharma S. Psychosocial characteristics associated with both antiretroviral therapy adherence and risk behaviors in women living with HIV. AIDS Behav. (2016) 20:1084–96. 10.1007/s10461-015-1209-5 26452670 PMC4826632

[20] Adeniyi OV Ajayi AI Selanto-Chairman N Ter Goon D Boon G Fuentes YO . Demographic, clinical and behavioural determinants of HIV serostatus nondisclosure to sex partners among HIV-infected pregnant women in the Eastern Cape, South Africa. PLoS ONE. (2017) 12:8. 10.1371/journal.pone.0181730 28837563 PMC5570311

[21] Kinuthia J Ronen K Unger JA Jiang W Matemo D Perrier T . SMS messaging to improve retention and viral suppression in prevention of mother-to-child HIV transmission (PMTCT) programs in Kenya: a 3-arm randomized clinical trial. PLoS Med. (2021) 18:5. 10.1371/journal.pmed.1003650 34029338 PMC8186790

[22] Yotebieng M Thirumurthy H Moracco KE Edmonds A Tabala M Kawende B . Conditional cash transfers to increase retention in PMTCT care, antiretroviral adherence, and postpartum virological suppression: a randomized controlled trial. JAIDS. (2016) 72:S124–S9. 10.1097/QAI.0000000000001062 27355499 PMC5113245

[23] Kiweewa FM Wabwire D Nakibuuka J Mubiru M Bagenda D Musoke P . Noninferiority of a task-shifting HIV care and treatment model using peer counselors and nurses among ugandan women initiated on art: evidence from a randomized trial. JAIDS. (2013) 63:E125–E32. 10.1097/QAI.0b013e3182987ce6 23807157

[24] Safren SA O'Cleirigh CM Bullis JR Otto MW Stein MD Pollack MH. Cognitive behavioral therapy for adherence and depression (CBT-AD) in HIV-infected injection drug users: a randomized controlled trial. J Consult Clin Psychol. (2012) 80:404–15. 10.1037/a0028208 22545737 PMC3365619

[25] Omonaiye O Nicholson P Kusljic S Manias E A. meta-analysis of effectiveness of interventions to improve adherence in pregnant women receiving antiretroviral therapy in sub-Saharan Africa. Int J Infect Dis. (2018) 74:71–82. 10.1016/j.ijid.2018.07.004 30003952

[26] Kim MH Ahmed S Tembo T Sabelli R Flick R Yu X . VITAL start: video-based intervention to inspire treatment adherence for life-pilot of a novel video-based approach to HIV counseling for pregnant women living with HIV. AIDS Behav. (2019) 23:3140–51. 10.1007/s10461-019-02634-1 31410618 PMC6803103

[27] Farmer KC. Methods for measuring and monitoring medication regimen adherence in clinical trials and clinical practice. Clin Ther. (1999) 21:1074–90. 10.1016/S0149-2918(99)80026-5 10440628

[28] Spinelli MA Haberer JE Chai PR Castillo-Mancilla J Anderson PL Gandhi M. Approaches to objectively measure antiretroviral medication adherence and drive adherence interventions. Curr HIV/AIDS Rep. (2020) 17:301–14. 10.1007/s11904-020-00502-5 32424549 PMC7363551

[29] Gebretsadik GG Gebretnsae H Ftwi M Tesfahunegn A. Alarm Clock-Based reminder for improving low adherence on option B plus antiretroviral therapy among HIV positive pregnant and lactating mothers in Northern Ethiopia. HIV AIDS-Res Palliative Care. (2020) 12:687–95. 10.2147/HIV.S261420 33177885 PMC7652568

[30] Wesevich A Mtande T Saidi F Cromwell E Tweya H Hosseinipour M . Role of male partner involvement in ART retention and adherence in Malawi's Option B plus program. AIDS Care - Psychol Socio-Med Asp. (2017) 29:1417–25. 10.1080/09540121.2017.1308464 28355926 PMC6372085

[31] Farley J Hines S Musk A Ferrus S Tepper V. Assessment of adherence to antiviral therapy in HIV-infected children using the Medication Event Monitoring System, pharmacy refill, provider assessment, caregiver self-report, and appointment keeping. J Acquir Immune Defic Syndr. (2003) 33:211–8. 10.1097/00126334-200306010-00016 12794557

[32] UNAIDS. 90-90-90: An Ambitious Treatment Target To Help End The Aids Epidemic. Available online at: https://www.unaids.org/en/resources/909090 (accessed September 5, 2022).

[33] Moher D Liberati A Tetzlaff J Altman DG. Preferred reporting items for systematic reviews and meta-analyses: the PRISMA statement. Int J Surg. (2010) 8:336–41. 10.1016/j.ijsu.2010.02.007 20171303

[34] Osterberg L Blaschke T. Drug therapy - adherence to medication. N Engl J Med. (2005) 353:487–97. 10.1056/NEJMra050100 16079372

[35] Higgins JP Altman DG Gøtzsche PC Jüni P Moher D Oxman AD . The Cochrane Collaboration's tool for assessing risk of bias in randomised trials. BMJ (Clinical research ed). (2011) 343:d5928. 10.1136/bmj.d5928 22008217 PMC3196245

[36] Guyatt G Oxman AD Akl EA Kunz R Vist G Brozek J . GRADE guidelines: 1. Introduction-GRADE evidence profiles and summary of findings tables. J Clin Epidemiol. (2011) 64:383–94. 10.1016/j.jclinepi.2010.04.026 21195583

[37] Balshem H Helfand M Schuenemann HJ Oxman AD Kunz R Brozek J . GRADE guidelines: 3 rating the quality of evidence. J Clin Epidemiol. (2011) 64:401–6. 10.1016/j.jclinepi.2010.07.015 21208779

[38] Maran BM Burey A. Matos TdP Loguercio AD Reis A. In-office dental bleaching with light vs without light: a systematic review and meta-analysis. J Dentistry. (2018) 70:1–13. 10.1016/j.jdent.2017.11.007 29289725

[39] Downs SH Black N. The feasibility of creating a checklist for the assessment of the methodological quality both of randomised and non-randomised studies of health care interventions. J Clin Epidemiol. (1998) 52:377–84. 10.1136/jech.52.6.377 9764259 PMC1756728

[40] Shi X-Q Wang Z-Z. Application of meta-regression and subgroup analyses of heterogeneity disposal in meta-analysis. Zhonghua Liu Xing Bing Xue Za Zhi. (2008) 29:497–501. Available online at: https://www.webofscience.com/wos/alldb/full-record/MEDLINE:18956686 18956686

[41] Nance N Pendo P Masanja J Ngilangwa DP Webb K Noronha R . Short-term effectiveness of a community health worker intervention for HIV-infected pregnant women in Tanzania to improve treatment adherence and retention in care: a cluster-randomized trial. PLoS ONE. (2017) 12:8. 10.1371/journal.pone.0181919 28859083 PMC5578486

[42] Weiss SM Peltzer K Villar-Loubet O Shikwane ME Cook R Jones DL. Improving PMTCT uptake in rural South Africa. J Int Assoc Provid AIDS Care. (2014) 13:269–76. 10.1177/2325957413488203 23778240 PMC4724424

[43] Okonji JA Zeh C Weidle PJ Williamson J Akoth B Masaba RO . CD4, Viral load response, and adherence among antiretroviral-naive breast-feeding women receiving triple antiretroviral prophylaxis for prevention of mother-to-child transmission of HIV in Kisumu, Kenya. JAIDS. (2012) 61:249–57. 10.1097/QAI.0b013e318262514f 22692094

[44] Mepham S Zondi Z Mbuyazi A Mkhwanazi N Newell ML. Challenges in PMTCT antiretroviral adherence in northern KwaZulu-Natal, South Africa. AIDS Care - Psychol Socio-Med Asp. (2011) 23:741–7. 10.1080/09540121.2010.516341 21293987

[45] Kieffer MP Nhlabatsi B Mahdi M Hoffman HJ Kudiabor K Wilfert CM. Improved detection of incident HIV infection and uptake of PMTCT services in labor and delivery in a high HIV prevalence setting. JAIDS. (2011) 57:E85–91. 10.1097/QAI.0b013e31821acc6e 21436709

[46] Cheng L Jiannv H Xiaohong Y Xianying C. Application of intelligent electronic medicine kits in medication compliance of HIV infected pregnant women with HAART. Chin J Aids & STD. (2020) 2:148–50+54. 10.13419/j.cnki.aids.2020.02.10

[47] Kanters S Park JJH Chan K Socias ME Ford N Forrest JI . Interventions to improve adherence to antiretroviral therapy: a systematic review and network meta-analysis. Lancet Hiv. (2017) 4:E31–40. 10.1016/S2352-3018(16)30206-5 27863996

[48] Cao W Hsieh E Li T. Optimizing treatment for adults with HIV/AIDS in China: successes over two decades and remaining challenges. Curr HIV/AIDS Rep. (2020) 17:26–34. 10.1007/s11904-019-00478-x 31939111 PMC6989417

[49] Lippman SA Shade SB El Ayadi AM Gilvydis JM Grignon JS Liegler T . Attrition and opportunities along the HIV care continuum: findings from a population-based sample, North West Province, South Africa. J Acquir Immune Defic Syndr. (2016) 73:91–9. 10.1097/QAI.0000000000001026 27082505 PMC4981507

[50] Wayne PM Lee MS Novakowski J Osypiuk K Ligibel J Carlson LE . Tai Chi and Qigong for cancer-related symptoms and quality of life: a systematic review and meta-analysis. J Can Survivorship. (2018) 12:256–67. 10.1007/s11764-017-0665-5 29222705 PMC5958892

[51] Rathbun RC Farmer KC Stephens JR Lockhart SM. Impact of an adherence clinic on behavioral outcomes and virologic response in the treatment of HIV infection: a prospective, randomized, controlled pilot study. Clin Ther. (2005) 27:199–209. 10.1016/j.clinthera.2005.02.010 15811483

[52] Naar-King S Outlaw AY Sarr M Parsons JT Belzer M MacDonell K . Motivational enhancement system for adherence (MESA): pilot randomized trial of a brief computer-delivered prevention intervention for youth initiating antiretroviral treatment. J Pediatr Psychol. (2013) 38:638–48. 10.1093/jpepsy/jss132 23359664 PMC3701125

[53] DiIorio C McCarty F Resnicow K Holstad MM Soet J Yeager K . Using motivational interviewing to promote adherence to antiretroviral medications: a randomized controlled study. AIDS Care - Psychol Socio-Med Asp. (2008) 20:273–83. 10.1080/09540120701593489 18351473 PMC3103182

[54] Ying S Xiaoning L Kejing H Jing W Jianwei L Bo W . Social support status and its correlation with medication adherence in pregnant women with HIV/AIDS. Chin JAids & STD. (2020) 26, 1190–2. 10.13419/j.cnki.aids.2020.11.12

[55] Hall RL Willgoss T Humphrey LJ Kongsø JH. The effect of medical device dose-memory functions on patients' adherence to treatment, confidence, and disease self-management. Patient Prefer Adherence. (2014) 8:775–88. 10.2147/PPA.S61248 24920889 PMC4043803

[56] Sabin LL DeSilva MB Gill CJ Zhong L Vian T Xie W . Improving adherence to antiretroviral therapy with triggered real-time text message reminders: the china adherence through technology study. JAIDS. (2015) 69:551–9. 10.1097/QAI.0000000000000651 25886927 PMC4552400

[57] Earnshaw VA Bogart LM Laurenceau J-P Chan BT Maughan-Brown BG Dietrich JJ . Internalized HIV stigma, ART initiation and HIV-1 RNA suppression in South Africa: exploring avoidant coping as a longitudinal mediator. J Int AIDS Soc. (2018) 21:10. 10.1002/jia2.25198 30362662 PMC6202800

[58] Kebede M Zeleke A Asemahagn M Fritz F. Willingness to receive text message medication reminders among patients on antiretroviral treatment in North West Ethiopia: A cross-sectional study. BMC Medical Inform Decis Mak. (2015) 15, 65. 10.1186/s12911-015-0193-z 26268394 PMC4535252

[59] Organization WH. Consolidated Guidelines on the Use of Antiretroviral Drugs for Treating and Preventing HIV Infection: Recommendations for a Public Health Approach 2016. Available online at: https://www.who.int/publications/i/item/9789241549684 (accessed July 12, 2021).

[60] Pellowski JA Price DM Harrison AD Tuthill EL Myer L Operario D . A systematic review and meta-analysis of antiretroviral therapy (ART) adherence interventions for women living with HIV. AIDS Behav. (2019) 23:1998–2013. 10.1007/s10461-018-2341-9 30443806 PMC6520201

[61] Liu J Xue Y Gao M Guo L. Psychological experience of pregnant women infected with HIV- a qualitative study. Chin J Modern Nurs. (2014) 26:3361–4. Available online at: https://kns.cnki.net/kcms/detail/detail.aspx?dbcode=CJFD&dbname=CJFDZHYX&filename=HLJH201426027&uniplatform=NZKPT&v=D5epmHu5xy0d1tvpR33X9wxGTfpRqpwb9Pq1eouF28B_5I18b1WWn6Qp7xhDcL3s24902487

[62] Peters H Thorne C Tookey PA Byrne L. National audit of perinatal HIV infections in the UK, 2006-2013: what lessons can be learnt? HIV Med. (2018) 19:280–9. 10.1111/hiv.12577 29336508 PMC5901012

[63] Vermeire E Hearnshaw H Van Royen P Denekens J. Patient adherence to treatment: three decades of research. A comprehensive review. J Clin Pharm Ther. (2001) 26:331–42. 10.1046/j.1365-2710.2001.00363.x 11679023

[64] Vanderpoel DR Hussein MA Watson-Heidari T Perry A. Adherence to a fixed-dose combination of rosiglitazone maleate/metformin hydrochloride in subjects with type 2 diabetes mellitus: a retrospective database analysis. Clin Ther. (2004) 26:2066–75. 10.1016/j.clinthera.2004.12.018 15823770

[65] Kromdijk W Mulder JW Rosing H Smit PM Beijnen JH Huitema ADR. Use of dried blood spots for the determination of plasma concentrations of nevirapine and efavirenz. J Antimicrob Chemother. (2012) 67:1211–6. 10.1093/jac/dks011 22302563

[66] Andrade SE Kahler KH Frech F Chan KA. Methods for evaluation of medication adherence and persistence using automated databases. Pharmacoepidemiol Drug Saf. (2006) 15:565–74. 10.1002/pds.1230 16514590

[67] Lavsa SM Holzworth A Ansani NT. Selection of a validated scale for measuring medication adherence. J Am Pharm Assoc. (2011) 51:90–4. 10.1331/JAPhA.2011.09154 21247831

[68] Ong CW Lee EB Twohig MP A. meta-analysis of dropout rates in acceptance and commitment therapy. Behav Res Ther. (2018) 104:14–33. 10.1016/j.brat.2018.02.004 29477890

