# Supplementary material for: Interventions to improve antiretroviral adherence in HIV-infected pregnant women: A systematic review and meta-analysis
Source: Front Public Health. 2022 Dec 8;10:1056915. doi: 10.3389/fpubh.2022.1056915 (PMC9773995; doi:10.3389/fpubh.2022.1056915)
Supplement: Supplementary file 1 [file Table_1.DOCX]

## Table S1. Database search strategies

| **Database and search date** | **Number of items found** |
| --- | --- |
| PubMed 31 May 2021 | 1140 |
| Web of Science 31 May 2021 | 1861 |
| Cochrane Central Register of Controlled Trials 31 May 2021 | 301 |
| Embase 1June 2021 | 872 |
| Cumulative index to Nursing and Allied Health Literature 2 June 2021 | 446 |
| **Total** | 4620 |

## PubMed 31 May 2021

| **Search** | **Term** | **Comments** |
| --- | --- | --- |
| **1.** | (((((((((((((((HIV Infections[MeSH Terms]) OR (HIV[MeSH Terms])) OR (hiv[Title/Abstract])) OR (hiv-1*[Title/Abstract])) OR (hiv-2*[Title/Abstract])) OR (hiv1[Title/Abstract])) OR (hiv2[Title/Abstract])) OR (hiv infect*[Title/Abstract])) OR (human immunodeficiency virus[Title/Abstract])) OR (human immuno-deficiency virus[Title/Abstract])) OR (human immune-deficiency virus[Title/Abstract])) OR (acquired immunodeficiency syndrome[Title/Abstract])) OR (acquired immuno-deficiency syndrome[Title/Abstract])) OR (acquired immune-deficiency syndrome[Title/Abstract])) OR (sexually transmitted diseases, viral[MeSH Terms])) OR (AIDS) | HIV/AIDS terms |
| **2.** | (acquired immun*) AND (deficiency syndrome[Title/Abstract]) |  |
| **3.** | (human immun*) AND (deficiency virus[Title/Abstract]) |  |
| **4.** | 1 OR 2 OR 3 |  |
| **5.** | ((((((((((pregnancy[MeSH Terms]) OR (pregnant[MeSH Terms])) OR (gestation[MeSH Terms])) OR (pregnan*[Title/Abstract])) OR (expected mother[Title/Abstract])) OR (mother[Title/Abstract])) OR (matern*[Title/Abstract])) OR (prenatal*[Title/Abstract])) OR (perinatal*[Title/Abstract])) OR (antenatal*[Title/Abstract])) OR (gestat*[Title/Abstract]) | Pregnancy terms |
| **6.** | 4 AND 5 |  |
| **7.** | (((((((Antiretroviral Therapy, Highly Active[MeSH Terms]) OR (Anti‐HIV Agents[Mesh] OR antiretroviral)) OR (antiretroviral[Title/Abstract])) OR (anti-retroviral[Title/Abstract])) OR (antiretroviral therapy[Title/Abstract])) OR (HARRT[Title/Abstract])) OR (highly active antiretroviral therapy[Title/Abstract])) OR (ART[Title/Abstract]) | ART terms |
| **8.** | ((((((Compliance[MeSH Terms]) OR (Patient compliance[MeSH Terms])) OR (Medication adherence[MeSH Terms])) OR (patient compliance[Title/Abstract])) OR (client compliance[Title/Abstract])) OR (participant compliance[Title/Abstract])) OR (adherence[Title/Abstract]) | Adherence terms |
| **9.** | 6 AND 7 AND 8 |  |

## Web of Science 31 May 2021

| **Search** | **Term** | **Comments** |
| --- | --- | --- |
| **1.** | TS=(HIV Infections OR HIV OR hiv OR hiv-1* OR hiv-2* OR hiv1 OR hiv2 OR hiv infect* OR human immunodeficiency virus OR human immuno-deficiency virus OR human immune-deficiency virus OR acquired immunodeficiency syndrome OR acquired immuno-deficiency syndrome OR acquired immune-deficiency syndrome OR sexually transmitted diseases, viral OR AIDS) | HIV/AIDS terms |
| **2.** | TS=(acquired immun* AND deficiency syndrome) |  |
| **3.** | TS=(human immun* AND deficiency virus) |  |
| **4.** | #3 OR #2 OR #1 |  |
| **5.** | TS=(pregnancy OR pregnant OR gestation OR pregnan* OR expected mother OR mother OR matern* OR prenatal* OR perinatal* OR antenatal* OR gestat*) | Pregnancy terms |
| **6.** | #5 AND #4 |  |
| **7.** | TS=(Antiretroviral Therapy, Highly Active OR Anti‐HIV Agents OR antiretroviral OR anti-retroviral OR antiretroviral therapy OR HARRT OR highly active antiretroviral therapy OR ART) | ART terms |
| **8.** | TS=(Compliance OR Patient compliance OR Medication adherence OR patient compliance OR client compliance OR participant compliance OR adherence) | Adherence terms |
| **9.** | #8 AND #7 AND #6 |  |

## Cochrane Central Register of Controlled Trials 31 May 2021

| **Search** | **Term** | **Comments** |
| --- | --- | --- |
| **1.** | MeSH descriptor: [HIV] this term only | HIV/AIDS terms |
| **2.** | MeSH descriptor: [HIV Infections] this term only |  |
| **3.** | MeSH descriptor: [Sexually Transmitted Diseases, Viral] this term only |  |
| **4.** | (hiv-1* OR hiv-2* OR hiv1 OR hiv2 OR hiv infect* OR human immunodeficiency virus OR human immuno-deficiency virus OR human immune-deficiency virus OR acquired immunodeficiency syndrome OR acquired immuno-deficiency syndrome OR acquired immune-deficiency syndrome OR AIDS):ti,ab,kw |  |
| **5.** | (acquired immun* AND deficiency syndrome):ti,ab,kw |  |
| **6.** | (human immun* AND deficiency virus):ti,ab,kw |  |
| **7.** | {or #1-#6} |  |
| **8.** | MeSH descriptor: [Pregnancy] this term only | Pregnancy terms |
| **9.** | (pregnan* or expected mother or mother OR matern* or prenatal* or perinatal* or antenatal* or gestat*):ti,ab,kw |  |
| **10.** | #8 or #9 |  |
| **11.** | #7 and #10 |  |
| **12.** | MeSH descriptor: [Antiretroviral Therapy, Highly Active] this term only | ART terms |
| **13.** | MeSH descriptor: [Anti-HIV Agents] this term only |  |
| **14.** | (antiretroviral OR anti-retroviral OR antiretroviral therapy OR HARRT OR highly active antiretroviral therapy OR ART):ti,ab,kw |  |
| **15.** | {or #12-#14} |  |
| **16.** | MeSH descriptor: [Compliance] this term only | Adherence terms |
| **17.** | MeSH descriptor: [Patient Compliance] this term only |  |
| **18.** | MeSH descriptor: [Medication Adherence] this term only |  |
| **19.** | (patient compliance OR client compliance OR participant compliance OR adherence):ti,ab,kw |  |
| **20.** | {or #16-#19} |  |
| **21.** | #11 and #15 and #20 |  |

## Cumulative index to Nursing and Allied Health Literature 2 June 2021

| **Search** | **Term** | **Comments** |
| --- | --- | --- |
| **1.** | MH( HIV Infections or HIV ) OR TI ( hiv or hiv-1* or hiv-2* or hiv1 or hiv2 or hiv infect* or human immunodeficiency virus or human immuno-deficiency virus or acquired immunodeficiency syndrome or acquired immuno-deficiency syndrome ) OR AB ( hiv or hiv-1* or hiv-2* or hiv1 or hiv2 or hiv infect* or human immunodeficiency virus or human immuno-deficiency virus or acquired immunodeficiency syndrome or acquired immuno-deficiency syndrome ) OR MH sexually transmitted diseases, viral OR AIDS | HIV/AIDS terms |
| **2.** | TI acquired immun* OR AB acquired immun* |  |
| **3.** | TI deficiency syndrome OR AB deficiency syndrome |  |
| **4.** | (S2 AND S3) |  |
| **5.** | TI human immun* OR AB human immun* |  |
| **6.** | TI deficiency virus OR AB deficiency virus |  |
| **7.** | (S5 AND S6) |  |
| **8.** | (S1 OR S4 OR S7) |  |
| **9.** | MH ( pregnancy or pregnant or gestation ) OR TI ( pregnan* or expected mother or mother or matern* or prenatal* or perinatal* or antenatal* or gestat* ) OR AB ( pregnan* or expected mother or mother or matern* or prenatal* or perinatal* or antenatal* or gestat* ) | Pregnancy terms |
| **10.** | (S8 AND S9) |  |
| **11.** | MH ( Antiretroviral Therapy, Highly Active or Anti‐HIV Agents or antiretrovira ) OR TI ( antiretroviral or anti-retroviral or antiretroviral therapy or HARRT or highly active antiretroviral therapy or ART ) OR AB ( antiretroviral or anti-retroviral or antiretroviral therapy or HARRT or highly active antiretroviral therapy or ART ) | ART terms |
| **12.** | MH ( compliance or patient compliance or medication adherence ) OR TI ( patient compliance or client compliance or participant compliance or adherence ) OR AB ( patient compliance or client compliance or participant compliance or adherence ) | Adherence terms |
| **13.** | (S10 AND S11 AND S12) |  |

## EMBase 1 June 2021

| **Search** | **Term** | **Comments** |
| --- | --- | --- |
| **9.** | #6 AND #7 AND #8 | **Final terms** |
| **8.** | 'compliance'/exp OR 'patient compliance'/exp OR 'medication adherence'/exp OR 'patient compliance':ab,ti OR 'client compliance':ab,ti OR 'participant compliance':ab,ti OR adherence:ab,ti | Adherence terms |
| **7.** | 'antiretroviral therapy, highly active'/exp OR 'anti‐hiv agents' OR antiretroviral:ab,ti OR 'anti retroviral':ab,ti OR 'antiretroviral therapy':ab,ti OR harrt:ab,ti OR 'highly active antiretroviral therapy':ab,ti OR art:ab,ti | ART terms |
| **6.** | #4 AND #5 | **Population terms** |
| **5.** | 'pregnancy'/exp OR 'pregnant' OR 'gestation'/exp OR pregnan*:ab,ti OR 'expected mother':ab,ti OR mother:ab,ti OR matern*:ab,ti OR prenatal*:ab,ti OR perinatal*:ab,ti OR antenatal*:ab,ti OR gestat*:ab,ti | Pregnancy terms |
| **4.** | #1 OR #2 OR #3 |  |
| **3.** | 'human immun*' AND 'deficiency virus':ab,ti |  |
| **2.** | 'acquired immun*' AND 'deficiency syndrome':ab,ti |  |
| **1.** | ((((((('hiv infectious' OR 'hiv' OR hiv:ab,ti OR 'hiv 1*':ab,ti OR 'hiv 2*':ab,ti OR hiv1:ab,ti OR hiv2:ab,ti OR 'hiv'/exp OR hiv) AND infect*:ab,ti OR 'human'/exp OR human) AND ('immunodeficiency'/exp OR immunodeficiency) AND virus:ab,ti OR 'human'/exp OR human) AND 'immuno deficiency' AND virus:ab,ti OR 'human'/exp OR human) AND ('immune deficiency'/exp OR 'immune deficiency') AND virus:ab,ti OR acquired) AND ('immunodeficiency'/exp OR immunodeficiency) AND syndrome:ab,ti OR acquired) AND 'immuno deficiency' AND syndrome:ab,ti OR acquired) AND ('immune deficiency'/exp OR 'immune deficiency') AND syndrome:ab,ti OR 'sexually transmitted diseases, viral'/exp OR 'sexually transmitted diseases, viral' OR 'aids'/exp OR aids | HIV/AIDS terms |

## Table S2 Meta regression

The results of meta-regression were presented below and none of the covariates are statistically meaningful. However, the number of included studies in our meta-analysis is less than 10, which means the result of regression is unstable ^[1]^. In this case, we have conducted several subgroups analyses to identify heterogeneity between groups preliminarily and the results of subgroups analyses suggest that measures and study settings might be the reasons of heterogeneity.

| **Subgroups** | **Coef.** | **SE** | **t** | **p-value** | **95%CI** | |
| --- | --- | --- | --- | --- | --- | --- |
| 2015 and later |  |  |  |  |  |  |
| Before 2015 | 0.277 | 0.342 | 0.81 | 0.504 | -1.196 | 1.749 |
| DBS |  |  |  |  |  |  |
| Pill counts | -0.148 | 0.364 | -0.41 | 0.724 | -1.713 | 1.417 |
| Medication possession ratio | -0.194 | 0.364 | -0.53 | 0.648 | -1.762 | 1.375 |
| Electronic monitoring systems | -0.381 | 0.336 | -1.14 | 0.373 | -1.823 | -1.060 |
| Quasi-experimental study |  |  |  |  |  |  |
| RCT | 0.254 | 0.336 | 0.76 | 0.528 | -1.190 | 1.698 |
| Rural |  |  |  |  |  |  |
| Urban | 0.307 | 0.339 | 0.90 | 0.461 | -1.151 | 1.764 |
| Cons | -0.125 | 0.349 | -0.36 | 0.754 | -1.626 | 1.375 |

[1] Shi Xiuquan, Wang Zengzhen. Application of Meta-regression and subgroup analyses of heterogeneity disposal in Meta-analysis. Chinese journal of epidemiology. 2008,29(5):497-501.
